# Newborn Screening for Sickle Cell Disease: Indian Experience

**DOI:** 10.3390/ijns4040031

**Published:** 2018-11-13

**Authors:** Roshan B. Colah, Pallavi Mehta, Malay B. Mukherjee

**Affiliations:** ICMR-National Institute of Immunohaematology, KEM Hospital Campus, Mumbai 400012, India

**Keywords:** newborn screening, sickle cell disease, India, tribal, non-tribal, Guthrie spots, cord blood, automated HPLC

## Abstract

Sickle cell disease (SCD) is a major public health problem in India with the highest prevalence amongst the tribal and some non-tribal ethnic groups. The clinical manifestations are extremely variable ranging from a severe to mild or asymptomatic condition. Early diagnosis and providing care is critical in SCD because of the possibility of lethal complications in early infancy in pre-symptomatic children. Since 2010, neonatal screening programs for SCD have been initiated in a few states of India. A total of 18,003 babies have been screened by automated HPLC using either cord blood samples or heel prick dried blood spots and 2944 and 300 babies were diagnosed as sickle cell carriers and SCD respectively. A follow up of the SCD babies showed considerable variation in the clinical presentation in different population groups, the disease being more severe among non-tribal babies. Around 30% of babies developed serious complications within the first 2 to 2.6 years of life. These pilot studies have demonstrated the feasibility of undertaking newborn screening programs for SCD even in rural areas. A longer follow up of these babies is required and it is important to establish a national newborn screening program for SCD in all of the states where the frequency of the sickle cell gene is very high followed by the development of comprehensive care centers along with counselling and treatment facilities. This comprehensive data will ultimately help us to understand the natural history of SCD in India and also help the Government to formulate strategies for the management and prevention of sickle cell disease in India.

## 1. Introduction

Hemoglobinopathies are the most common monogenic disorders in India posing a significant health burden. Sickle cell disease (SCD) was the first molecular disease to be described where a single point mutation (A→T) resulted in the substitution of the 6th aminoacid in the β globin chain from glutamic acid to valine leading to an altered electrophoretic mobility of the hemoglobin molecule. SCD includes a variety of conditions, the primary hemoglobin disorder being sickle cell anemia (SCA) due to homozygosity for hemoglobin S (HbS) as well as the compound heterozygous conditions, HbS-β thalassemia, HbSD disease, HbSE disease, HbSC disease and HbS-O Arab disease [1].

## 2. Geographic Distribution of HbS in India

HbS is widespread in African, Mediterranean, Middle Eastern, Indian, Caribbean and South and Central American populations [1]. In India, SCA is prevalent among tribal populations who are considered to be the original inhabitants in south Gujarat, Maharashtra, Madhya Pradesh, Chhattisgarh, and western Odisha with a smaller focus in the southern region in Andhra Pradesh, Karnataka, northern Tamil Nadu and Kerala. They often reside in remote regions away from the mainstream populations. Sickle cell anemia is also prevalent in some of the scheduled castes and other backward classes (non-tribal populations) mainly in central India, in particular, among the Mahar, Kunbi and Teli castes but is rare in other castes where it may be seen due to admixture. These are economically and socially disadvantaged populations living largely in rural areas. Carrier frequencies ranging from 1 to 35% have been described in these groups and their distribution in different states of the country has been mapped earlier [2,3,4]. Many of these populations also harbor the β thalassemia gene but screening for β thalassemia had not been extensively carried out among them earlier. A recent report from Akola district in central India showed that 36 out of 91 pediatric SCD patients (39.6%) had sickle-β thalassemia [5].

## 3. Sickle Haplotypes in India

In the eastern province of Saudi Arabia and in India, the sickle gene is linked to the Arab Indian or Asian haplotype, which is associated with higher fetal hemoglobin (HbF) levels and a milder clinical presentation than the Benin haplotype seen in west and north Africa and the Bantu haplotype seen in east and central Africa [6]. There are limited studies on haplotype analysis from different regions in India, however, all of them have shown that around 90% of sickle genes have the Asian haplotype while few other atypical haplotypes including the BantuA2 and the Senegal haplotype have been associated with around 10% of sickle genes [7]. Yet, recent studies have indicated more severe clinical manifestations even among the Asian haplotype.

## 4. Clinical Manifestations of Sickle Cell Disease in India

Initial studies on sickle cell disease patients from western Odisha demonstrated a mild clinical course with higher hemoglobin levels, lower reticulocyte counts, persistence of splenomegaly, infrequent leg ulcers and priapism compared to patients with the disease of African origin [8]. Subsequently, in western and central India it was found that the disease was milder among tribal populations in Valsad in south Gujarat compared to non-tribal populations in Nagpur in Maharashtra. Apart from higher HbF levels, a significant ameliorating factor was the presence of associated α thalassemia, which was very common in tribal populations in Gujarat [9]. Since then, reports of more severe features, particularly from central India have raised the question of geographic variations in the manifestations of SCD within India. In a retrospective study, where 316 children with sickle cell anemia were followed up for a period of 5.8 ± 5.7 years in Nagpur, there were 1725 hospitalizations among 282 patients and 96 children had severe disease with severe vaso occlusive crises, severe anemia, splenic sequestration, stroke and hypersplenism being reported and 10 babies died during this period [10]. Another retrospective analysis of 110 adult patients with sickle cell disease who attended out-patient clinics or were admitted to the hospital showed that 75.4% of them had a severe disease in spite of all the sickle genes being linked to the Asian haplotype and the presence of associated α thalassemia [11]. Thus, sickle cell disease has an extremely variable clinical presentation in Indian patients.

## 5. Providing Comprehensive Care in Rural Regions

As a large majority of sickle cell disease cases are born in rural India, it is necessary to provide adequate care for these patients, which can be challenging in remote areas. However, few studies from different regions have demonstrated that this is possible with the involvement of Government and Non-Government Organizations as well as representatives from the local population. It was shown in a study from the Bardoli district in Gujarat that giving basic health care training to a local villager to regularly visit households and monitor sickle cell disease cases and identify those with significant complications and refer them to the coordinating hospital would have a significant impact on tribal communities with a high prevalence of SCD [12]. In another study undertaken in a remote tribal village in Gudalur in south India, it was shown that 71% of 111 patients with sickle cell disease were able to have at least one annual comprehensive care visit. Premature deaths were seen in 19 patients at a median age of 23 years due to acute chest syndrome, sepsis, severe anemia, stroke or sudden unexplained deaths [13]. A comprehensive care model at Sewa Rural in Gujarat was successfully implemented, which included screening, both out-patient and in-patient care of SCD patients as well as health education. A one year follow up of 164 SCD patients in this rural region was possible and pain crises were seen in 72 patients (43.9%); 59 patients (35.9%) required hospitalization, 43 patients (26.2%) required blood transfusions and three patients (1.6%) died during this short follow up [14].

## 6. Benefits of Newborn Screening and Comprehensive Care

Newborn screening (NBS) enables the identification of babies with sickle cell disease at birth or soon after, within the first few days of their life before they present with any symptoms or complications. These babies can then be regularly followed up with the provision of comprehensive care and timely management to reduce morbidity and mortality. It has been demonstrated in several countries that early diagnosis and providing care is critical in SCD because of the possibility of lethal complications in the first few years of life in pre-symptomatic children. Young children with SCD have an increased susceptibility to bacteremia due to *Streptococcus pneumonia*, which can be fatal in many cases. Acute splenic sequestration crisis is another cause for mortality in infancy. Many studies done globally have shown that early prophylactic penicillin can significantly reduce the morbidity and mortality due to pneumococcal sepsis and they have also shown the importance of pneumococcal vaccines for the prevention of pneumococcal sepsis, thus justifying the need for implementing newborn screening programs for SCD [15,16].

## 7. Newborn Screening Initiatives in India

**Figure 1:**
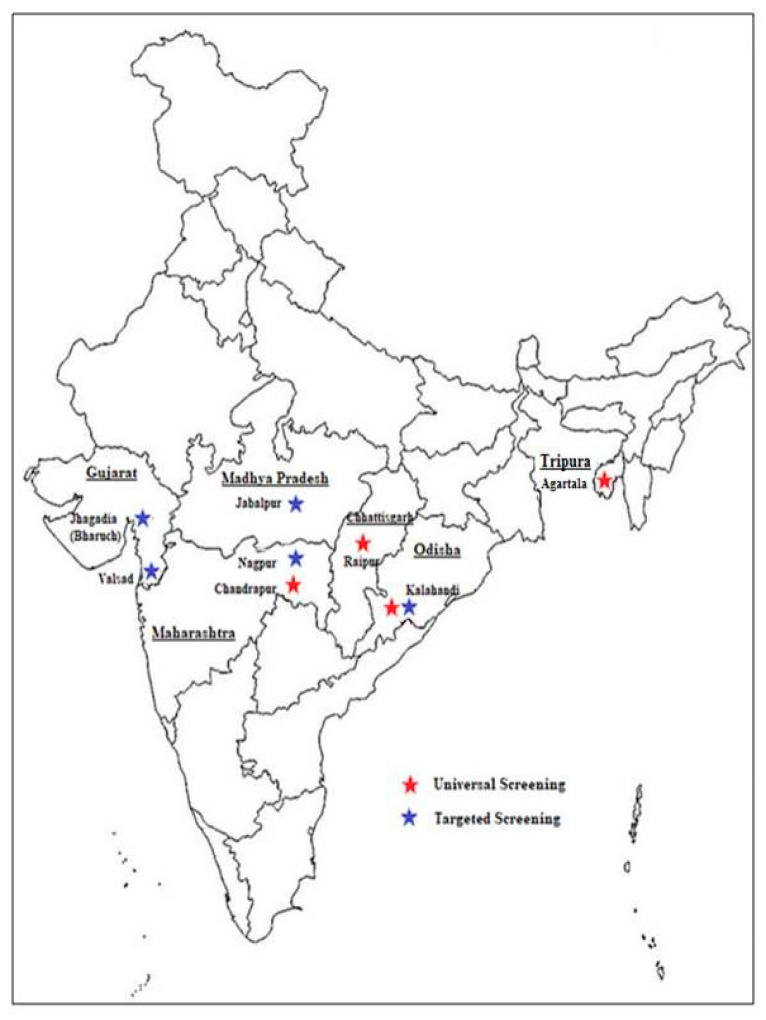
Location of the different centers in India where newborn screening was undertaken.

It has been estimated that three countries, Nigeria, D R Congo and India are most affected by SCD and widespread newborn screening and follow up care could save the lives of almost 10 million children by 2050. It is also estimated that 15% of the world’s neonates with sickle cell anemia are born in India [17]. Thus, newborn screening has a great relevance in this country. There is no National neonatal screening program for SCD as yet and affected children are generally identified when they become symptomatic. However, few newborn screening programs have been initiated in some regions in the last 5 to 6 years. Figure 1 shows the location of the different centers involved in newborn screening programs in India. Table 1 summarizes the programs undertaken in a few states in this vast country.

Most of the above were pilot studies undertaken in different states, which showed that it was feasible to undertake newborn screening for SCD even in rural regions and register affected babies for follow up and comprehensive care although the outcome of the follow up was not reported in all these studies.

## 8. Technologies Used for Newborn Screening in India

Globally, isoelectric focusing (IEF) using eluates from dried blood spots was initially used for screening of newborn babies for sickle cell disease but at many centers this had been replaced by high-performance liquid chromatography (HPLC) analysis [24,25]. In all of the Indian reports on newborn screening for SCD, HPLC analysis has been used. This was mainly due to two reasons. Automated HPLC machines were already in use at these centers for other programs, hence, no additional cost for infrastructure was required. Secondly, it was felt that these machines would be easier to operate and maintain even in rural areas. The Variant NBS machine (BioRad laboratories, Hercules, CA, USA) has been used for hemoglobin analysis from dried blood spots or the Variant Hemoglobin Testing System (BioRad laboratories) for cord blood samples using either the sickle cell short or the β thal short programs. The β thal short program had the advantage of picking up other hemoglobin abnormalities including some rare non-deletional α chain variants like Hb Fontainebleau, Hb O Indonesia and Hb Koya Dora [26]. More recently, several point-of-care devices have been developed for screening, which are either paper-based screening protocols or antibody-based rapid diagnostic devices based on lateral flow immunoassay technologies. They are simple to use and relatively inexpensive as they do not require any specific equipment or even electricity, which is often not always available in remote rural regions. These commercial devices are still being validated for newborn screening for SCD [27,28]. Presently, we are also undertaking a multicenter validation of one of these newborn screening kits to evaluate its suitability for use in newborn screening programs in India in the future.

## 9. Follow up of Birth Cohorts of Sickle Cell Disease in India

A systematic follow up of SCD babies for around 4 to 5 years had been possible in at least two newborn screening programs in the country in Valsad in south Gujarat and Nagpur in Maharashtra [18,19]. The program in Gujarat targeted mainly tribal newborn babies from four districts. These babies were largely from nine different tribes, the highest numbers being from the Dhodia Patel, Kukna and Halpita tribes. It involved 13 centers (government district hospitals to community health centers) for neonatal sample collection on filter paper. These dried blood spots were sent for processing on the NBS variant machine to the centralized laboratory in Valsad. Of the 5467 babies screened, 687 were identified as sickle cell trait and 46 babies had sickle cell disease (SS-33, S-β-thal-13). After confirmation of the diagnosis, the SCD babies were registered for comprehensive care. Follow up from 1.5 to 5 years was possible only in 32 (69%) of these babies. Pneumococcal vaccination and folic acid supplementation were given to all of the babies. In this cohort, 18 babies (SS-11, S-β thal-7) had no clinical complications till the last follow-up. The majority of the babies who became symptomatic presented after 2 years of age. Seven babies (SS-6, S-β thal-1) had severe complications, which included severe infections, vasoocclusive crises, severe anemia and acute chest syndrome. Few others had mild febrile episodes and mild splenomegaly and hepatomegaly were seen in some babies. One baby died at the age of 4 years during the follow up period. Although haplotyping was not done in all of the SS babies, the Xmn1 polymorphism in 24 SS babies, where this was determined, was Xmn1 (+/+) in all of them. The HbF levels varied from 12.5 to 30.2% among those SS babies who were between 2.4 and 5 years of age. The prevalence of α thalassemia was 92% in this population, the most common α genotype being −α^3.7^/−α^3.7^ [18]. In the second phase of this program, mobile phones were given to the parents of affected babies to improve compliance for follow ups and this had a significant impact.

In Nagpur in Maharashtra, newborn screening was done at a single government medical college where only babies born to sickle heterozygous mothers were screened making the program more cost-effective. The population here was largely non-tribal, the majority being from the Mahar community. A total of 2134 babies of mothers having a positive solubility test were screened by the collection of cord blood at birth or a heel prick sample subsequently and analyzed by HPLC on the Variant Hemoglobin Testing System. There were 978 babies with sickle cell trait and 113 babies with sickle cell disease (SS-104, S-βthal-7, SD-2). In this cohort too, 73% of the babies could be followed up for 3 to 5 years. Penicillin prophylaxis was given to the babies who could be followed up. Here several babies presented much earlier than the cohort in Gujarat and 45% of the SCD babies required hospitalization between 3 months and 2 years. Infections, severe anemia and painful events were the common presenting features. Eight sickle homozygous babies had sepsis. Six SCD babies died during the follow up period. Haplotyping was done in 75 SS babies and 141 of the 150 SS chromosomes (94%) were linked to the Asian haplotype. Six SS chromosomes were linked to the Bantu A2 haplotype and three to an atypical haplotype. The mean HbF level in the SS cohort was 21.4 ± 5.4%. The prevalence of α thalassemia in this cohort was 28%, the −α^3.7^/αα genotype being the commonest defect.

Newborn screening for hemoglobinopathies was also undertaken in the malaria endemic northeastern region in Agartala in Tripura where Hb E is widely prevalent but Hb S is also seen among the tea garden workers who are migrant laborers from other states [23]. Only 15 newborn babies with sickle cell trait were identified but 9.3% of babies had HbE trait, 3.3% were Hb E homozygous and one baby had HbE-β thalassemia. Screening for G6PD deficiency was also done and few babies with Hb abnormalities were also G6PD deficient.

The clinical presentation among sickle cell disease babies was quite variable in the two cohorts which could be followed up for at least 4 to 5 years. As mentioned in a recent editorial, the question remains whether the intervention programs developed for African disease could be applied to Indian patients with the Asian haplotype [29]. Newborn cohort studies in different regions in India will be able to answer these questions once they have been systematically undertaken and the affected babies followed up for a longer duration.

## 10. Lessons Learnt from Pilot Studies on Newborn Screening for Sickle Cell Disease in India

The feasibility of establishing newborn screening programs in tribal areas in rural regions has been shown. Follow up of birth cohorts in the studies where this was done showed that the clinical presentation was very variable in different regions. Further efforts and motivation are needed to ensure that the maximum number of babies can be enrolled and continue to receive comprehensive care and the follow up of babies can be done for a longer duration. Newborn screening programs must be extended to other states where the sickle gene is prevalent. Guidelines for a National Hemoglobinopathy Program have been recently laid down by the Ministry of Health and Family Welfare which also includes newborn screening for SCD and these will be followed for understanding the natural history of sickle cell disease in India [30].

## Figures and Tables

**Table 1:** Summary of newborn screening programs initiated in India.

State	District	Target Population	Sample	Technology for Screening	No. Screened	No(%)AS	No(%)SCD	Follow Up	Reference
South Gujarat Phase 1	Valsad	All Tribal babies	Heel prick-Dried blood spot	HPLC-Variant NBS machine	5467	687 (12.5%)	46 (0.8%)	5–6 years	Italia et al., 2015 [18]
South Gujarat Phase 2	Valsad, Bharuch	All Tribal babies	Heel prick-Dried blood spot	HPLC-Variant NBS machine	2944	649 (22.0%)	76 (2.6%)	2 years	Unpublished
Maharashtra	Nagpur	Largely non-tribal, babies of AS mothers	Cord blood, heel prick	HPLC-Variant Hb Testing System	2134	978 (45.8%)	113 (5.3%)	4-5 years	Upadhye et al., 2016 [19]
Madhya Pradesh	Jabalpur	Tribal, babies of AS mothers	Cord blood, heel prick	HPLC-Variant Hb Testing System	461	36 (7.8%)	6 (1.3%)	1 year	Unpublished
Chhattisgarh	Raipur	Tribal and non-tribal babies	Heel prick-Dried blood spot	HPLC-Variant NBS machine	1158	61 (5.3%)	6 (0.5%)	No follow up reported	Panigrahi et al., 2012 [20]
Odisha	Kalahandi	Tribal and non-tribal babies	Heel prick-Dried blood spot	HPLC-Variant Hb Testing System	1668	293 (17.6%)	34 (2.0%)	No follow up reported	Mohanty et al., 2010 [21]
Odisha	Kalahandi	Tribal babies	Cord blood	HPLC-Variant Hb Testing System	761	112 (14.7%)	13 (1.7%)	No follow up reported	Dixit et al., 2015 [22]
Tripura	Agartala	Tribal & non tribal babies	Cord blood	HPLC-Variant Hb Testing System	2400	15 (0.6%)	0 (0.0%)	Not done	Upadhye et al., 2018 [23]
Maharashtra	Chandrapur	Tribal and non-tribal babies	Cord blood, heel prick	HPLC-Variant Hb Testing System	1010	85 (8.4%)	4 (0.4%)	Not done	Unpublished
